# Gut microbiota dysbiosis and metabolic perturbations of bile/glyceric acids in major depressive disorder with IBS comorbidity

**DOI:** 10.1128/mbio.02447-25

**Published:** 2025-10-07

**Authors:** Jia-Yu Du, Zhen-Jie Zhang, Li Tan, Jing-Yi Yang, Run-Nan Yang, Yi-Long Chen, Gui-Feng Tan, Jing Li, Wen-Jing Li, Lin Yang, Jia Cai, Dan-Lin Shen, Hong-Ru Zhu, Zhen-Xin Fan, Min-Lan Yuan, Wei Zhang

**Affiliations:** 1 Mental Health Center, West China Hospital, Sichuan University 617943, Chengdu, Sichuan, China; 2 Key Laboratory of Bioresources and Ecoenvironment (Ministry of Education), College of Life Sciences, Sichuan University 162677, Chengdu, China; 3 West China Biomedical Big Data Center, West China Hospital, Sichuan University 34753, Chengdu, China; Georgia Institute of Technology, Atlanta, Georgia, USA

**Keywords:** major depressive disorder, irritable bowel syndrome, microbiota, metabolite, gut-brain axis

## Abstract

Major depressive disorder (MDD) and irritable bowel syndrome (IBS) exhibit high comorbidity, yet their shared pathophysiology remains unclear. Previous studies have primarily focused on the psychological health in the IBS population, without considering psychiatric diagnoses or stratifying different psychological states, potentially leading to biased findings. This study employed multi-omics approaches to characterize gut microbiota and serum metabolites in 120 MDD patients (47 with IBS and 73 without IBS) and 70 healthy controls (HCs). MDD with IBS patients showed significantly higher depression (Hamilton depression scale [HAMD-17]) and anxiety (Hamilton anxiety scale [HAMA-14]) scores than MDD-only patients (*P* < 0.05). Metagenomic sequencing of fecal samples revealed increased alpha diversity (Chao1/Shannon indices) and *Firmicutes* dominance in both MDD groups vs HC, while *Actinobacteria* enrichment specifically marked MDD with IBS. Functionally, MDD with IBS uniquely activated D-amino acid/glycerolipid metabolism pathways (Kyoto Encyclopedia of Genes and Genomes). Serum metabolomics identified comorbid-specific perturbations: downregulation of bile acids (CDCA, GCDCA, GCDCA-3S) and upregulation of glyceric acid/glutaconic acid. Our study also found that *Eggerthella lenta* and *Clostridium scindens* are differentially abundant bacteria that are involved in bile acid metabolism, and that microbial genes (e.g., K03738) are associated with glyceric acid production. These findings implicate gut microbiota-driven bile acid/glyceric acid dysregulation in MDD with IBS comorbidity, supporting the gut-brain axis as a therapeutic target for probiotics or microbiota transplantation.

**IMPORTANCE:**

Major depressive disorder (MDD) exhibits a high comorbidity rate with irritable bowel syndrome (IBS). Our study, conducted on 120 MDD patients (47 of whom were comorbid with IBS) and a control group of 70 individuals, revealed that MDD-IBS comorbid patients demonstrated significantly higher depression/anxiety scores. Multi-omics analysis indicated substantial alterations in the gut microbiota (e.g., *Firmicutes*, *Actinobacteria*) and serum metabolites (e.g., bile acids, glyceric acid) among MDD-IBS patients, which were associated with specific metabolic pathways. Therefore, the new aspect of this study was the inclusion of patients with MDD but without IBS symptoms, which provided a deeper understanding of the intestinal microbiota dysregulation associated with comorbid IBS and MDD. These findings suggest that there may be involvement of the gut-brain axis, providing new research directions for potential therapeutic targets.

**CLINICAL TRIALS:**

This study is registered with the Chinese Clinial Trial Registry as ChiCTR2100041598.

## INTRODUCTION

Major depressive disorder (MDD), a mental illness characterized by persistent low mood, anhedonia or loss of interest, and significant fatigue or excessive tiredness, affects approximately 300 million individuals worldwide (1). It is currently ranked as the third leading cause of global disease burden according to the World Health Organization (2008), with projections suggesting it will become the primary contributor by 2030. Irritable bowel syndrome (IBS) is a chronic gastrointestinal disorder characterized by abdominal pain or discomfort along with alterations in stool frequency or consistency (2). The comorbidity rate between MDD/anxiety disorders and IBS ranges from 44% to 84% (3). Notably, patients with comorbid MDD and IBS often suffer from poorer quality of life, suboptimal treatment outcomes, and more severe symptoms (4). Moreover, individuals with IBS face an elevated risk for subsequent development of MDD, anxiety disorders, sleep disturbances, and bipolar disorder (5).

One potential explanation for the association between MDD and IBS is their shared etiology, such as psychological stress, which has been implicated in the onset and severity of both MDD (6) and IBS (7). Previous studies have primarily focused on the psychological health in the IBS population without considering psychiatric diagnoses (8, 9), thereby potentially introducing bias due to a lack of a control group of MDD alone without IBS.

Recently, there has been a growing body of research focusing on the alterations in intestinal microbiota observed in patients with MDD or IBS (10–13). For instance, studies have highlighted changes in the abundance of *Actinobacteria* and *Bacteroidota* among individuals with MDD (14, 15), as well as variations in the levels of *Firmicutes* and *Bacteroidota* among those with IBS (11, 16). Notably, one study discovered similarities between the fecal microbiota composition of MDD and IBS patients (11). However, limited research has been conducted to investigate the gut microbiota in patients with comorbid MDD and IBS using metagenomic sequencing and associated function. Given that intestinal microorganisms play crucial roles in vital physiological processes such as nutrient absorption, substance metabolism, and immune defense mechanisms, they are closely linked to various disease occurrences (17–19). One contributing factor is their impact on metabolites that enter systemic circulation. The interactions between gut microbiota and their hosts encompass a diverse array of crucial metabolites, including microbial metabolites generated through bacterial fermentation of dietary substances, host molecules modified by bacteria, or products directly synthesized by bacteria (20). For instance, altering the composition of the microbial community may influence the occurrence of diseases by regulating the composition of bile acids (BAs) (21). Short-chain fatty acids (SCFAs) (such as acetic acid, propionic acid, and butyric acid) are formed through the bacterial fermentation of dietary fibers, and different bacteria (such as *Lactobacilli* and *Bacteroides*) produce specific SCFAs to regulate energy metabolism and intestinal barrier function (22). Moreover, carnitine has multiple microbial metabolic pathways, while indole derived from tryptophan mediates communication between the intestine and the brain (23). In summary, these metabolites emphasize the importance of the gut-microbiota-brain axis. Hence, an increasing number of studies have now focused on alterations in the gut microbiota composition among patients with MDD, leading to changes in the microbial metabolomes that contribute to the pathogenesis of MDD (24–26).

Therefore, to elucidate how IBS comorbidity influences MDD progression through microbial and metabolic pathways, we conducted a comprehensive multi-omics analysis. We integrated psychological assessments, metagenomic sequencing of gut microbiota, and serum metabolomic profiling in rigorously diagnosed MDD patients with and without IBS. We aim to (i) identify comorbid-specific microbial and metabolic signatures, (ii) uncover functional links between gut microbiota dysbiosis and host metabolite dysregulation, and (iii) provide mechanistic insights for developing gut-targeted therapies to alleviate both gastrointestinal and psychiatric symptoms.

## MATERIALS AND METHODS

### Subjects

**Fig 1:**
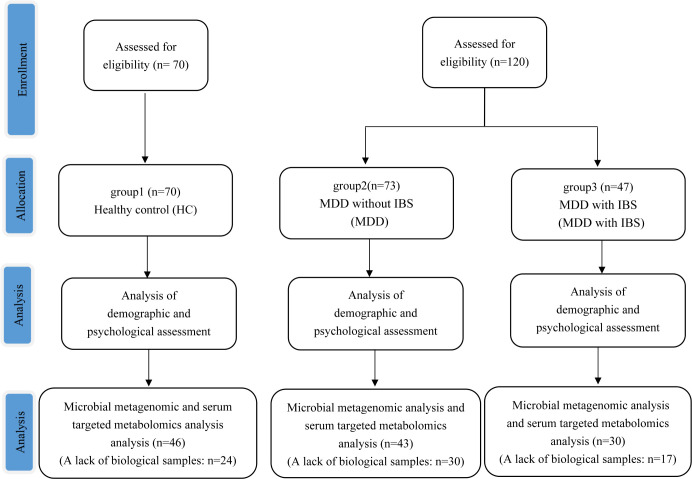
Flow chart diagram of subject enrollment and analysis.

From October 2021 to August 2023 at the Mental Health Center of West China Hospital, Sichuan University, we enrolled 120 patients diagnosed with MDD using the Mini International Neuropsychiatric Interview (27), assessed by senior psychiatrists. In addition, we recruited 70 healthy subjects without MDD and IBS as the control group (healthy control [HC]). To be eligible for participation in this study, participants had to meet the following criteria: (i) age between 18 and 65 years (inclusive), (ii) education level of primary school or higher with a sufficient understanding of the study content, (iii) voluntary consent to participate in the study after being informed about its purpose and procedures, and finally, (iv) no medication use for more than 2 weeks or intermittent medication use for less than 3 days within a 2-week period. However, individuals were excluded if they presented with severe physical illness, organic brain disease, neurological disorder, or cognitive impairment. Moreover, individuals with comorbid mental illnesses such as schizophrenia and bipolar disorder were also excluded. Furthermore, individuals who consumed probiotics daily prior to their arrival at our center were also excluded from participating in this study (Fig. 1).

### Assessment and biological sample collection

After arrival at our center, all subjects completed the following battery of tests: IBS Severity Scoring System (IBS-SSS) (28), Hamilton Depression Scale (HAMD-17) (29), Hamilton Anxiety Scale (HAMA-14) (30), Screening Depression Questionnaire (PHQ-9) (31), and Generalized Anxiety Disorder Scale (GAD-7) (32). The four scales used in this study (HAMA-14, GAD-7, PHQ-9, and HAMD-17) collectively constituted the participants’ emotional state.

The IBS-SSS employs a questionnaire to assess the severity of abdominal pain, frequency of abdominal pain, degree of abdominal distension, defecation patterns, and impact on patients' quality of life. Scores are assigned using a visual scoring method ranging from 0 to 500, with higher scores indicating more severe symptoms. Based on test scores, participants were categorized into three levels: mild (75–175 points), moderate (176–300 points), and severe (301–500 points). MDD patients with an IBS-SSS score <75 were classified as the MDD without IBS group (MDD); conversely, those with an IBS score ≥75 were classified as the MDD with IBS group (MDD with IBS).

Demographic data were also collected for all subjects, including age, sex, smoking, drinking, body mass index (BMI), religion, civil status, employment status, and monthly household income. Meanwhile, stool and serum samples were also collected on the day of subject enrollment. Among them, 71 participants lacked a biological sample (Fig. 1).

### Metagenomic sequencing and data processing

DNA was extracted from fecal samples, and the purity and integrity of the extracted DNA were assessed by agarose gel electrophoresis. DNA concentration was accurately quantified using a Qubit fluorometer (Thermo Fisher Scientific). Subsequently, library preparation was performed, which included DNA fragmentation, end repair, A-tailing, adapter ligation, purification, and PCR amplification. After library construction, library quality and fragment size distribution were evaluated using an Agilent 2100 Bioanalyzer and quantitative PCR (qPCR). High-throughput sequencing was conducted on the Illumina NovaSeq 6000 platform using a paired-end 150 bp (PE150) mode.

Raw sequencing data underwent quality control with fastp (v0.19.3), including removal of adapter contamination, low-quality reads, and reads shorter than the threshold, resulting in high-quality clean reads. Clean reads were then aligned to the human reference genome GRCh38 (RefSeq: GCF_000001405.40) using Bowtie2 (v2.3.4) to filter out host-derived contamination. The remaining non-host reads were assembled both individually and in a combined manner using MEGAHIT (v1.2.9). Assembly quality was assessed with QUAST, considering metrics such as N50, total number of contigs, and total assembly length. Contigs of length ≥ 500 bp were subjected to open reading frame prediction using MetaGeneMark (v3.38). Predicted genes were filtered, standardized in naming, and clustered at 95% sequence similarity using CD-HIT (v4.8.1) to generate a non-redundant gene catalog (Unigenes). Clean reads from each sample were mapped back to the Unigene sequences using Bowtie2 to obtain read counts per gene. Unigenes with mapped reads ≤2 across all samples were removed to produce a high-quality gene set for downstream analysis. For taxonomic annotation, the protein sequences corresponding to Unigenes were aligned against bacterial, fungal, archaeal, and viral protein sequences extracted from the NCBI NR database (version 2022.05) using DIAMOND (v6.24.20). Taxonomic classification was performed based on the lowest common ancestor algorithm implemented in MEGAN (v0.9.24), generating species abundance profiles from kingdom to species levels. For functional annotation, DIAMOND was used to align Unigene protein sequences against the Kyoto Encyclopedia of Genes and Genomes (KEGG) database (version 2022.05). For each sequence, the annotation with the highest bit score >60 was retained for subsequent KEGG pathway annotation and functional analyses.

### Targeted metabolomics analysis and data processing

All of the standards of targeted metabolites were obtained from Sigma-Aldrich (St. Louis, MO, USA), Steraloids Inc. (Newport, RI, USA), and TRC Chemicals (Toronto, ON, Canada). All the standards were accurately weighed and prepared in water, methanol, sodium hydroxide solution, or hydrochloric acid solution to obtain individual stock solution at a concentration of 5.0 mg/mL. An appropriate amount of each stock solution was mixed to create stock calibration solutions. Formic acid was of Optima grade and obtained from Sigma-Aldrich (St. Louis, MO, USA). Methanol (Optima LC-MS), acetonitrile (Optima LC-MS), and isopropanol (Optima LC-MS) were purchased from Thermo-Fisher Scientific (Fair Lawn, NJ, USA). Ultrapure water was produced by a Mill-Q Reference system equipped with an LC-MS Pak filter (Millipore, Billerica, MA, USA).

Serum samples were thawed in an ice bath to minimize degradation. A 20 µL aliquot of plasma was transferred into a 96-well plate, followed by the addition of 120 µL of ice-cold methanol solution containing partial internal standards. After vigorous vortexing for 5 min, the mixture was centrifuged at 4,000 *× g* for 30 min. The plate was then transferred to an Eppendorf epMotion Workstation (Eppendorf Inc., Hamburg, Germany). Subsequently, 30 µL of the supernatant was transferred to a new 96-well plate, and 20 µL of freshly prepared derivatization reagent (200 mM 3-NPH in 75% aqueous methanol and 96 mM EDC-6% pyridine solution in methanol) was added to each well. The plate was sealed and incubated at 30°C for 60 min to allow derivatization. After the reaction, 330 µL of ice-cold 50% methanol solution was added to each well for dilution. The plate was then placed at −20°C for 20 min, followed by centrifugation at 4,000 *× g* for 30 min at 4°C. Subsequently, 135 µL of the supernatant was transferred to a new 96-well plate preloaded with 10 µL of internal standard solution. Calibration was performed using derivatized standard mixtures of varying concentrations added to the wells on the left side of the plate. Quality control samples were prepared by pooling equal volumes from each individual sample. All samples were stored at −80°C until analysis. Quantitative analysis of all target metabolites was conducted using an ultra-performance liquid chromatography–tandem mass spectrometry (UPLC-MS/MS) system (Acquity UPLC-Xevo TQ-S, Waters Corp., Milford, MA, USA). Raw data generated from the UPLC-MS/MS were processed using TMBQ software (v1.0, Metabo-Profile, Shanghai, China) for peak integration, calibration, and quantification of each metabolite. Metabolite concentrations in unknown samples were determined by comparison with a series of standards of known concentrations via calibration curves. These calibration curves characterize the relationship between the analytical signal and analyte concentration. For most metabolites, the instrument response (e.g., peak height or peak area) exhibits a linear relationship with concentration, which can be described by the equation *y* = *ax* + *b*, where *y* represents the instrument response, *a* is the slope reflecting the method’s sensitivity, *b* accounts for the background signal, and *x* denotes the analyte concentration in the unknown sample. Using this model, metabolite concentrations in unknown samples were calculated from the measured instrument responses, enabling accurate and reproducible quantification.

In this study, we employed the Q300 kit (Metabo-Profile, Shanghai, China), an automated high-throughput metabolite array technology that enables quantitative detection of multiple metabolites across different concentration ranges within a single microtiter plate. This platform allows for absolute quantification as well as differential screening of diverse classes of metabolites, including amino acids, phenols, phenyl-derivatives or benzyl-derivatives, indoles, organic acids, fatty acids, carbohydrates, and bile acids. The kit incorporates isotope-labeled internal standards (e.g., L-arginine-15N2, hippuric acid-D5, TCDCA-D9, D-glucose-D7, carnitine-D3, C5:0-D9, and citric acid-D4), together with matched external standards, to ensure accurate qualitative and quantitative analyses (33). Of the more than 300 targeted metabolites, a total of 201 were successfully detected in our samples. The Q300 method provides broad coverage of disease-relevant metabolites, including those implicated in MDD and IBS, while offering high analytical accuracy, stability, and reproducibility.

### Statistical analysis

All analyses in this study were performed within the R software environment. Clinical data were statistically analyzed using the R package tableone (v0.13.2) (34) . Categorical variables were analyzed using the chi-square test, and continuous variables were compared between the two groups using the *t*-test. Multiple hypothesis testing was adjusted using the Benjamini–Hochberg (BH) method to control the false discovery rate. Microbial community composition analysis was conducted using the R package vegan (v2.6-10) (35). Community structure differences were assessed by calculating Bray–Curtis distance matrices, and significance testing was performed via permutation-based multivariate analysis of variance (PERMANOVA) implemented by the adonis2 function. Alpha diversity indices were also calculated using the same package. Differential species analysis was conducted with the R package microeco (v1.9.1) in conjunction with the linear discriminant analysis effect size (LEfSe) method to identify characteristic taxa, with selection criteria of linear discriminant analysis (LDA) score >2 and *P* value <0.05 (36). Pathway enrichment analysis based on KEGG Orthology (KO) relative abundances was performed using the R package ReporterScore (v0.1.9), combining Wilcoxon and Kruskal–Wallis tests with 999 permutations. Significantly enriched pathways were selected based on absolute Reporter scores >1.96 and visualized accordingly (37). Correlations between variables were assessed using Spearman’s rank correlation coefficients, with significance determined by corresponding *P* values.

To explore the co-variation among microbiome, serum metabolome, and psychological phenotypes, pairwise data sets were subjected to co-inertia analysis (CIA) using the R package made4 (v1.76.0) (38). After dimensionality reduction by principal component analysis (PCA), co-inertia structures were computed, and their significance was evaluated by permutation testing of the RV coefficient with 999 permutations. The results were visualized as arrow plots, illustrating sample trajectories across the two omics projection spaces, indicating synergistic variation. In the metabolomics analysis, the Wilcoxon rank-sum test was used to assess the significance between the two groups. PCA and orthogonal partial least squares discriminant analysis (OPLS-DA) were conducted for sample classification and modeling. Variable importance in projection (VIP) scores were subsequently calculated to evaluate the contribution of each metabolite to the model classification. Differential metabolites were identified based on the criteria of *P* value <0.05 and VIP > 1.

## RESULTS

A total of 190 participants were included for scale assessment and demographic analysis after screening. Among them, 70 patients belonged to the HC group, 73 patients belonged to the MDD group, and 47 patients belonged to the MDD with IBS group. Subsequently, due to insufficient biological samples from 71 patients, a total of 119 patients were finally included for further gut metagenomic analysis and serum targeted metabolomics analysis (HC: 46 participants; MDD: 43 patients; and MDD with IBS: 30 patients) (Fig. 1).

### Comorbid IBS aggravates anxiety and depression in patients with MDD

Among the 190 subjects, there were no statistically significant differences in age, sex, smoking status, alcohol consumption, BMI, religious affiliation, marital status, employment status, and monthly household income among the three groups (Table 1). The MDD group exhibited significantly higher scores on the IBS-SSS compared to the HC group. Furthermore, the MDD with IBS group demonstrated significantly elevated scores on the IBS-SSS as well as HAMD-17 and HAMA-14 scales when compared to the MDD group (Table 1). These findings suggest that our sampled MDD patients, those with comorbid IBS, had more pronounced levels of anxiety and depression.

**TABLE 1:** Comparison of characteristics among three groups (*n* = 190)^*a*^

Characteristic	HC (*n* = 70)	MDD (*n* = 73)	MDD with IBS (*n* = 47)	*P*	Adjust *P*_A-B_	Adjust *P*_A-C_	Adjust *P*_B-C_
Men	40 (57.14)	27 (36.99)	10 (21.28)	0.0526			
Age, yr	32.93 ± 10.36	35.30 ± 12.16	37.62 ± 14.70	0.1253			
Body mass index, kg/m^2^	22.87 ± 4.63	22.01 ± 4.05	21.75 ± 3.81	0.3029			
Self-reported religious beliefs	1 (1.43)	6 (8.22)	4 (8.51)	0.1445			
Civil status				0.1420			
Unmarried	42 (60.00)	32 (43.84)	20 (42.55)				
Married	27 (38.57)	37 (50.68)	23 (48.94)				
Divorced/widowed	1 (1.43)	4 (5.48)	4 (8.51)				
Employment				0.1093			
Unemployed	1 (1.43)	12 (16.44)	5 (10.64)				
Working full-time	41 (58.57)	33 (45.20)	22 (46.81)				
Working part-time	21 (30.00)	17 (23.29)	10 (21.28)				
Student	3 (4.29)	6 (8.22)	5 (10.64)				
Retired	4 (5.71)	3 (4.11)	3 (6.38)				
Housewife	0 (0.00)	2 (2.74)	2 (4.25)				
Monthly household income, USD^*b*^				0.4729			
<419.58	8 (11.43)	8 (10.96)	5 (10.64)				
419.58–839.16	18 (25.71)	13 (17.81)	6 (12.77)				
839.16–1,258.74	16 (22.86)	19 (26.03)	11 (23.40)				
1,258.74–1,678.32	8 (11.43)	11 (15.07)	13 (27.66)				
>1,678.32	20 (28.57)	22 (30.14)	12 (25.53)				
Current smoking				0.3314			
No	52 (74.28)	55 (75.34)	38 (80.85)				
Yes	17 (24.29)	14 (19.18)	9 (19.15)				
Previous smoking	1 (1.43)	4 (5.48)	0 (0.00)				
Current drinking				0.3035			
No	31 (44.28)	41 (56.16)	30 (63.83)				
Yes	37 (52.86)	31 (42.47)	16 (34.04)				
Previous drinking	2 (2.86)	1 (1.37)	1 (2.13)				
Psychological assessments							
IBS-SSS	18.04 ± 19.83	28.68 ± 20.48	145.4 ± 79.31	<0.0001	0.0095	<0.0001	<0.0001
HAMD-17	2.49 ± 2.13	17.67 ± 4.48	19.47 ± 4.60	<0.0001	<0.0001	<0.0001	0.0127
HAMA-14	1.80 ± 3.11	20.39 ± 6.69	23.64 ± 8.07	<0.0001	<0.0001	<0.0001	0.0048
PHQ-9	2.29 ± 3.17	17.28 ± 5.82	17.98 ± 4.88	<0.0001	<0.0001	<0.0001	0.7180
GAD-7	2.16 ± 3.28	12.47 ± 5.22	13.37 ± 5.00	<0.0001	<0.0001	<0.0001	0.5412

^*a*^ Values are presented as *n*, *n* (%), mean ± SD, or median (interquartile range), unless otherwise noted.

^*b*^ 1 USD = 7.15 CNY.

### Metagenomic analysis reveals alterations in gut microbiota in patients with MDD comorbid with IBS

**Fig 2:**
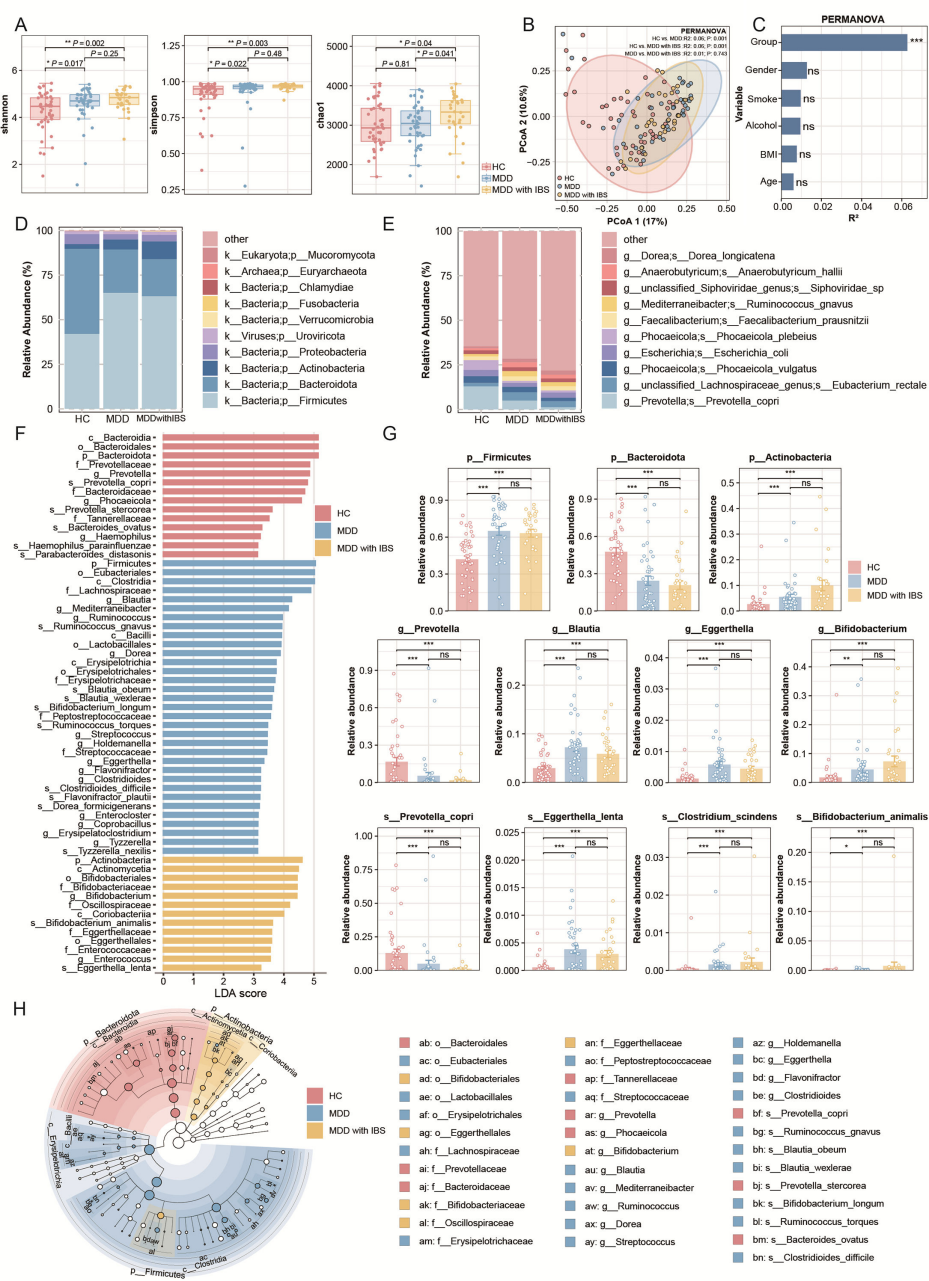
Metagenomic taxonomic profiling and microbial community differences among HC, MDD, and MDD with IBS groups. (**A**) Box plots of alpha diversity indices (Shannon, Simpson, and Chao1) at the species level, comparing microbial richness and evenness among groups. (**B**) Principal coordinates analysis (PCoA) based on Bray–Curtis distances of species-level microbial composition. Group differences were evaluated using PERMANOVA (adonis), with *R*² and *P* values shown for pairwise comparisons: HC vs MDD, HC vs MDD with IBS, and MDD vs MDD with IBS. (**C**) PERMANOVA of potential confounding factors. The *x*-axis represents the proportion of variance explained (*R*²), and the *y*-axis lists different confounders. Statistical significance: *P* > 0.05 (ns); *, *P* < 0.05; **, *P* < 0.01; ***, *P* < 0.001. (**D**) Stacked bar chart showing the average relative abundance of the top 10 bacterial phyla in each group, illustrating phylum-level microbial composition across HC, MDD, and MDD with IBS. (**E**) Stacked bar chart showing the average relative abundance of the top 10 bacterial species in each group, reflecting species-level differences in microbiota composition. (**F**) LEfSe analysis showing the top 60 discriminative taxa ranked by LDA score (LDA score > 2, *P* < 0.05). (**G**) LEfSe results highlighting representative phyla, genera, and species enriched in different groups, presented as bar plots with overlaid dots. Statistical significance: *P* > 0.05 (ns); *, *P* < 0.05; **, *P* < 0.01; ***, *P* < 0.001. (**H**) Radial cladogram illustrating the phylogenetic distribution of significantly enriched taxa identified by LEfSe, from phylum to species. Colors indicate the groups in which taxa are enriched (HC, MDD, or MDD with IBS), and concentric rings represent successive taxonomic ranks from phylum (innermost) to species (outermost). The letter labels correspond to the names shown within the concentric rings.

One-hundred nineteen subjects were included for gut metagenomic sequencing and analysis, including 46 HC, 43 MDD, and 30 MDD with IBS. The total sequencing data for each sample and the proportion of reads classified as microbial are summarized in Table S1. Alpha diversity, assessed by Shannon and Simpson indices, was significantly higher in both MDD and MDD with IBS groups compared to HC. The Chao1 index was further elevated in the MDD with IBS group, indicating increased microbial richness under comorbid conditions (*P* < 0.05) (Fig. 2A). Principal coordinates analysis and PERMANOVA showed significant differences in microbial community structure between the disease groups and the HC group (*P* < 0.05), while no significant differences were observed between the MDD and MDD with IBS groups (Fig. 2B). Further PERMANOVA at the species level showed that group status significantly affected gut microbial beta diversity (*R*² = 0.0627, *P* < 0.001) while sex, age, BMI, smoking, and alcohol consumption showed no significant effects (*P* > 0.05) (Fig. 2C). At the phylum level, *Firmicutes*, *Bacteroidota*, and *Actinobacteria* were dominant across all samples, with *Firmicutes* most enriched in the disease groups (Fig. 2D). At the species level, the top 10 dominant species included *Prevotella copri*, *Eubacterium rectale*, and *Phocaeicola vulgatus*, with the HC group showing the highest total relative abundance of these species (Fig. 2E). LEfSe analysis identified numerous significantly different microbial taxa across taxonomic levels (from phylum to species) between groups (Fig. 2F). At the phylum level, *Firmicutes* were enriched in the MDD group, *Bacteroidota* in HC, and *Actinobacteriota* in the MDD with IBS group. At another level, several signature microbes were differentially enriched in each group. For instance, *Prevotella copri* was enriched in HC, while *Clostridium scindens* and *Bifidobacterium animalis* were enriched in the MDD with IBS group (Fig. 2G). Figure 2H illustrates the phylogenetic relationships of significantly enriched taxa with relatively high abundance across groups. Pairwise LEfSe analyses revealed significant microbial differences between disease groups and HC, but not between MDD and MDD with IBS (Table S2).

### Functional gene and KEGG pathway analysis of fecal metagenomes reveals distinct differences between MDD and MDD with IBS groups

**Fig 3:**
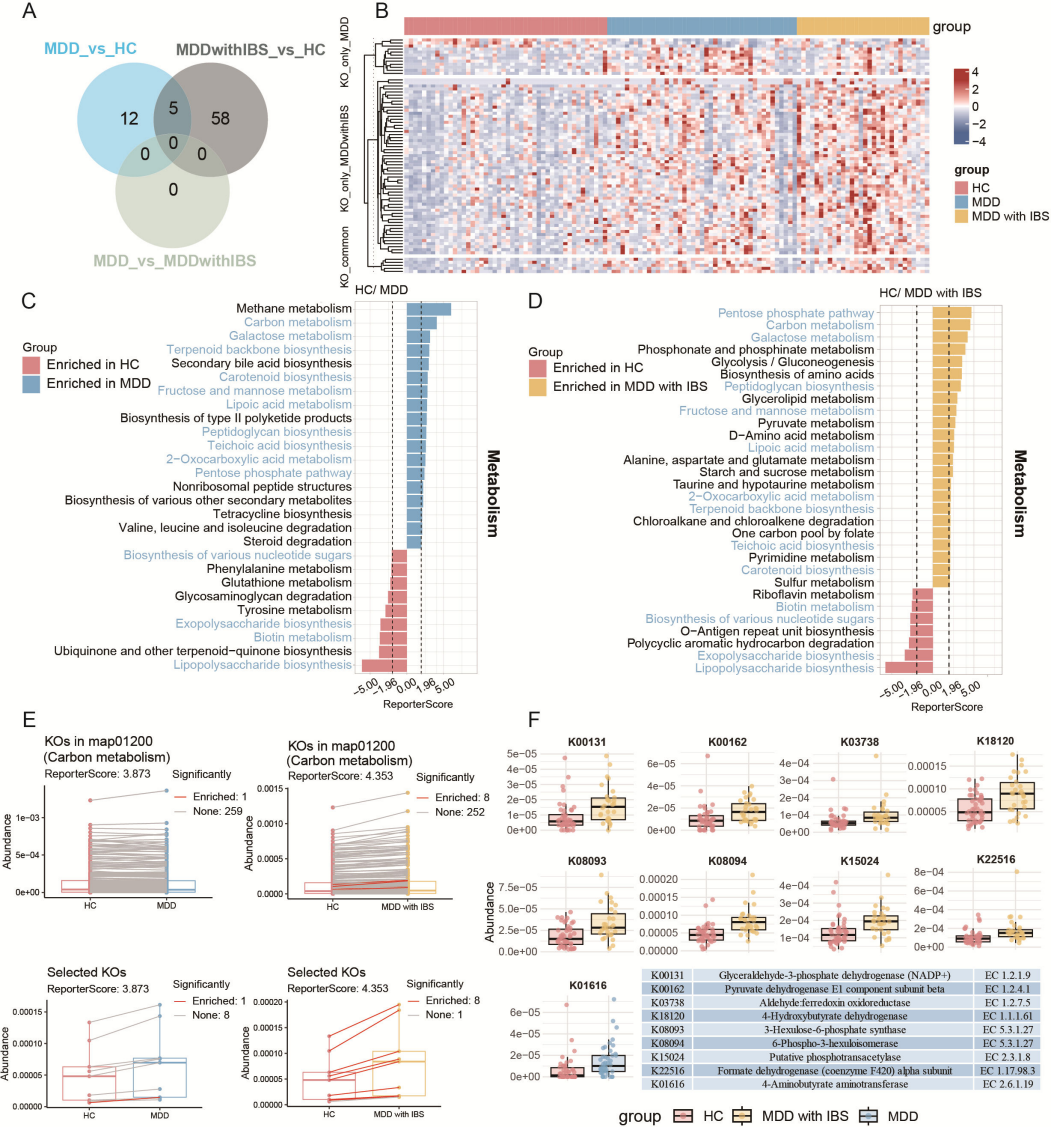
Functional differences in the gut microbiome between MDD and MDD with IBS at the KO and pathway levels. (**A**) Venn diagram showing the number of significantly different KOs among comparison groups. KO abundance was analyzed using the Wilcoxon rank-sum test with 999 permutations, and *P* values were adjusted by the BH method (*P* < 0.05). (**B**) Heatmap of significantly altered KOs across individual samples. (**C**) KEGG pathway enrichment analysis between HC and MDD groups based on reporter score (|ReporterScore| > 1.96). Shared pathways in panels C and D are highlighted in blue. (**D**) KEGG pathway enrichment analysis between HC and MDD with IBS groups based on reporter score (|ReporterScore| > 1.96). Pathway enrichment was conducted using the Generalized Reporter Score Analysis method. Only metabolic pathway enrichment results are shown here, while comprehensive enrichment results including other pathways are provided in the supplemental figures. (**E**) Summary abundance box plots of all significantly enriched KOs involved in the carbon metabolism pathway (map01200) in HC vs MDD (left) and HC vs MDD with IBS (right). The lower panels show the abundance of nine differential KOs (one in HC vs MDD; eight in HC vs MDD with IBS) involved in the carbon metabolism pathway (map01200) in HC vs MDD (left) and HC vs MDD with IBS (right). (**F**) Box plots with overlaid dot plots displaying the distribution of the nine differential KOs among HC vs MDD and HC vs MDD with IBS groups. A table in the figure lists the enzyme names and EC numbers for each KO.

At the KO level, more differential KOs were identified between the MDD with IBS and HC groups (*n* = 63) than between the MDD and HC groups (*n* = 17). There were five shared upregulated KOs in both disease groups compared to HC (Fig. 3A). Most KOs uniquely altered in the MDD with IBS group also showed an upward trend in MDD, though to a lesser extent (Fig. 3B). KEGG pathway enrichment analysis revealed significant enrichment in multiple pathways in both MDD and MDD with IBS groups (|ReporterScore| > 1.96). Specifically, 47 pathways were enriched in MDD vs HC and 83 pathways in MDD with IBS vs HC (Fig. S1). In metabolism pathways, shared pathways primarily included those involved in carbon metabolism (map01200), pentose phosphate pathway (map00030), and 2-oxocarboxylic acid metabolism (map01210), among others. The MDD with IBS group uniquely enriched pathways, including D-amino acid metabolism (map00470), glycerolipid metabolism (map00561), and others (Fig. 3C and D). Further analysis showed stronger upregulation of functional genes in shared pathways in the MDD with IBS group. For example, only the multifunctional 2-oxoglutarate metabolism enzyme (K01616) was significantly upregulated in MDD, while eight KOs were upregulated in MDD with IBS (Fig. 3E and F). Notably, most enriched pathways were significantly correlated with emotional state and IBS-SSS score (Fig. S2). Although pathway enrichment analysis showed significant results between the two disease groups, no significant KO-level differences were observed, and no further pathway analysis was performed. These results suggest the necessity of integrating metabolomics to better understand the impact of gut microbiota on metabolic processes.

### Serum targeted metabolomics has revealed the metabolic characteristics of the MDD with IBS group

**Fig 4:**
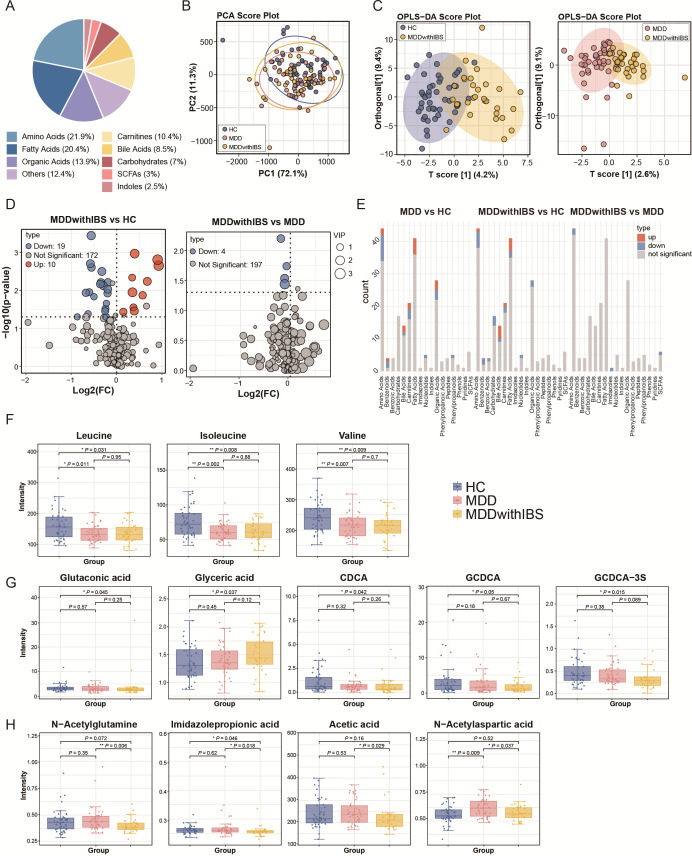
Multivariate statistical analysis and identification of differential metabolites across HC, MDD, and MDD with IBS groups. (**A**) Pie chart of detected metabolites by class. (**B**) PCA score plot of HC, MDD, and MDD with IBS groups. (**C**) OPLS-DA score plots: MDD with IBS vs HC (*R*^2^*X* = 0.136, *R*^2^*Y* = 0.62, *Q*^2^ = 0.109) and MDD with IBS vs MDD (*R*^2^*X* = 0.116, *R*^2^*Y* = 0.594, *Q*^2^ = −0.647). (**D**) Volcano plots of differential metabolites between MDD with IBS vs HC and MDD with IBS vs MDD. Metabolites with *P* < 0.05 and VIP > 1.0 are highlighted. Blue dots indicate downregulated metabolites, and red dots indicate upregulated metabolites. (**E**) Bar plot of metabolite classification. Blue bars represent downregulated metabolites, and red bars represent upregulated metabolites. (**F**) Box plots of metabolites significantly downregulated in both the MDD vs HC and MDD with IBS vs HC comparisons (leucine, isoleucine, and valine). Statistical significance: *, *P* < 0.05; **, *P* < 0.01; ***, *P* < 0.001. (**G**) Box plots of metabolites showing significant differences only in the MDD with IBS vs HC comparison (glutaconic acid, glyceric acid, CDCA, GCDCA, and GCDCA-3S). Statistical significance: *, *P* < 0.05; **, *P* < 0.01; ***, *P* < 0.001. (**H**) Box plots of metabolites significantly altered between the MDD with IBS and MDD groups (N-acetylglutamine, imidazolepropionic acid, acetic acid, and N-acetylaspartic acid). Statistical significance: *, *P* < 0.05; **, *P* < 0.01; ***, *P* < 0.001.

A total of 201 metabolites were detected across 119 serum samples (Table S3), and their classification is presented in Fig. 4A. PCA showed a significant difference between the MDD and HC groups (*P* = 0.044), whereas no significant differences were observed between the MDD with IBS and HC groups (*P* = 0.193) or between the MDD with IBS and MDD groups (*P* = 0.804) (Fig. 4B). Further analysis using an OPLS-DA model showed distinct clustering patterns among the groups (MDD with IBS vs HC: *R*²*X* = 0.136, *R*²*Y* = 0.62, *Q*² = 0.109; MDD with IBS vs MDD: *R*²*X* = 0.116, *R*²*Y* = 0.594, *Q*² = −0.647) (Fig. 4C). Based on the differential screening criteria (*P* < 0.05 and VIP > 1), multiple differential metabolites were identified. In the MDD with IBS group compared to the HC group, 10 metabolites were significantly upregulated and 19 were downregulated. Compared to the MDD group, only four metabolites were significantly downregulated in the MDD with IBS group (Fig. 4D). In addition, a total of nine metabolites showed significant differences exclusively in the MDD with IBS group.

We classified and analyzed the differential metabolites. The metabolite classification bar plot showed that most differential metabolites in the MDD with IBS group were derived from amino acids, fatty acids, carbohydrates, and bile acids (Fig. 4E). Analysis of the differential metabolites revealed that leucine, isoleucine, and valine were significantly downregulated in both the MDD vs HC and MDD with IBS vs HC comparisons. Meanwhile, glutaconic acid, glyceric acid, CDCA, GCDCA, and GCDCA-3S showed significant alterations only in the MDD with IBS vs HC comparison. Furthermore, four metabolites—N-acetylglutamine (NAG), imidazolepropionic acid, acetic acid, and N-acetylaspartic acid (NAA)—were significantly downregulated in the MDD with IBS vs MDD comparison. The box plots illustrate the relative abundance changes of these metabolites across the different groups (Fig. 4F through H).

### Correlation between gut microbiota and metabolites in the MDD with IBS group

**Fig 5:**
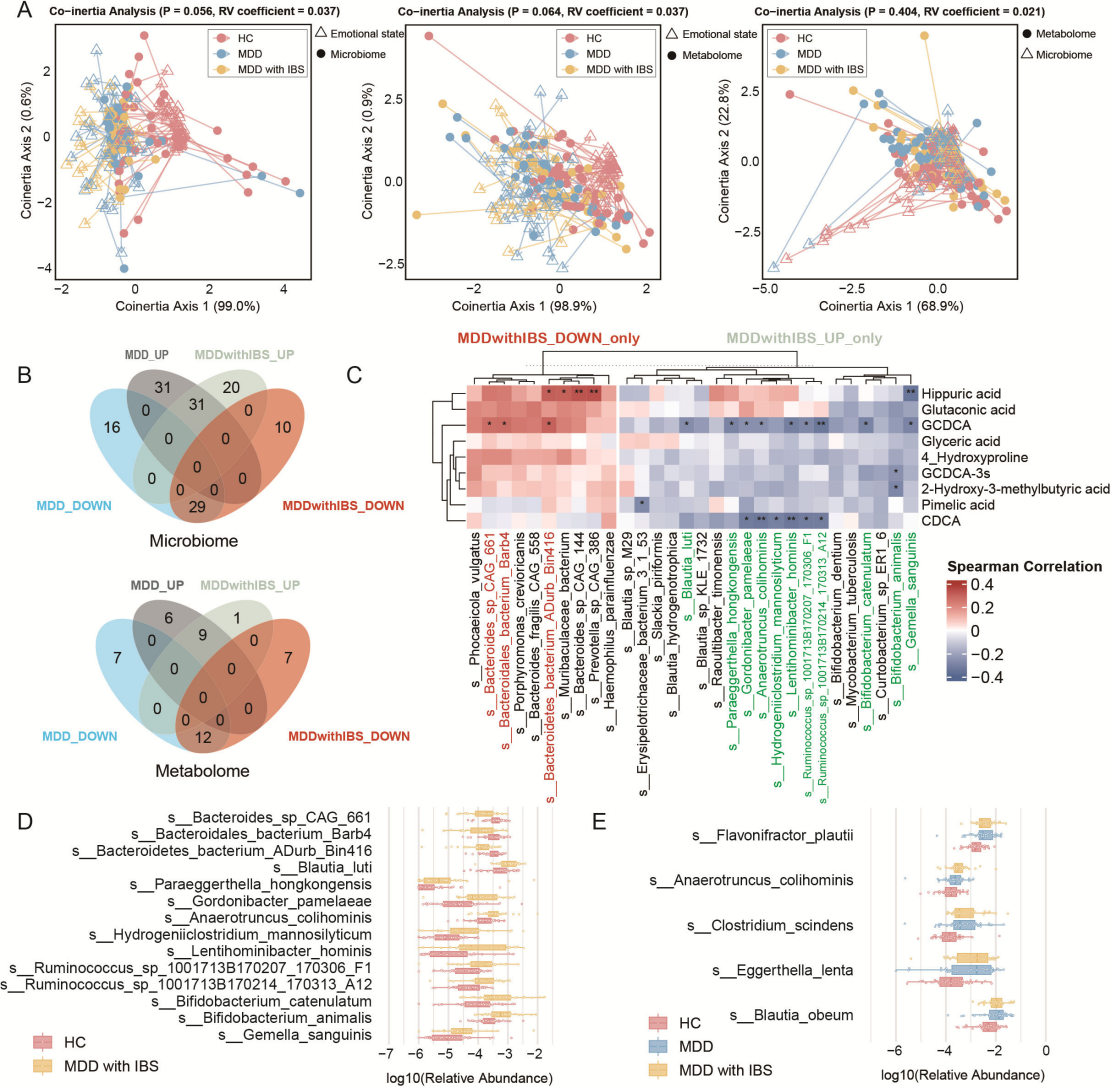
Correlation analysis between gut microbiota and serum metabolites in the MDD with IBS group. (**A**) CIA of pairwise relationships among cognitive status, gut microbiota composition, and serum metabolite profiles. Lines connect the two datasets from the same individual. (**B**) Venn diagrams showing differentially abundant features among groups. The top panel depicts microbial species identified by LEfSe analysis comparing HC vs MDD and HC vs MDD with IBS groups, with 20 enriched and 10 depleted taxa specific to the MDD with IBS group. The bottom panel shows differentially abundant metabolites in the same comparisons, with one enriched and seven depleted metabolites specific to the MDD with IBS group, highlighting features uniquely associated with comorbid IBS in MDD patients. (**C**) Spearman correlation heatmap between MDD with IBS-specific microbial species and differential metabolites. Statistical significance: *, *P* < 0.05; **, *P* < 0.01; ***, *P* < 0.001. Microbial species significantly associated with bile acid levels are highlighted in red and green. Red indicates species specifically downregulated in MDD with IBS, while green indicates species specifically upregulated in MDD with IBS. (**D**) Box plots with overlaid dot plots showing the log_10_ relative abundance of microbial species significantly associated with bile acid levels in the HC and MDD with IBS groups. (**E**) Box plots with overlaid dot plots showing the log_10_ relative abundance of representative bile acid–associated taxa that were differentially abundant in both HC vs MDD and HC vs MDD with IBS comparisons, and that have been previously reported to be involved in bile acid metabolism.

Using CIA across all samples, we examined the influence of emotional state on gut microbiota composition and serum metabolite levels, as well as their interaction. Emotional state showed a marginal effect on both data sets, but the overall covariation between microbiota and metabolites was not statistically significant (Fig. 5A). MDD with IBS-specific differential microbes was identified, including 20 enriched and 10 depleted taxa, while the specific differential metabolites included only one enriched metabolite (glyceric acid) and seven depleted metabolites (Fig. 5B; Tables S2 and S4). Spearman correlation analysis revealed significant associations between several altered species and bile acid levels. Upregulated taxa such as *Anaerotruncus colihominis*, *Lentihominibacter hominis*, and *Gordonibacter pamelaeae* were positively correlated with CDCA and GCDCA levels. Additionally, *Paraeggerthella hongkongensis* and *Blautia luti* were negatively correlated with GCDCA. Conversely, downregulated species like *Bacteroidetes bacterium ADurb Bin416* and *Bacteroidales bacterium Barb4* showed positive correlations with GCDCA. No significant correlations were observed between glyceric acid and the altered microbiota (Fig. 5C). The abundances of bile acid–associated taxa are shown in Fig. 5D. Notably, shared differentially abundant taxa in both the MDD and MDD with IBS groups, such as *Blautia obeum*, *Eggerthella lenta*, and *Clostridium scindens*, are known to be involved in bile acid metabolism (Fig. 5E).

### Functional annotation and differential metabolite analysis in the MDD with IBS group

**Fig 6:**
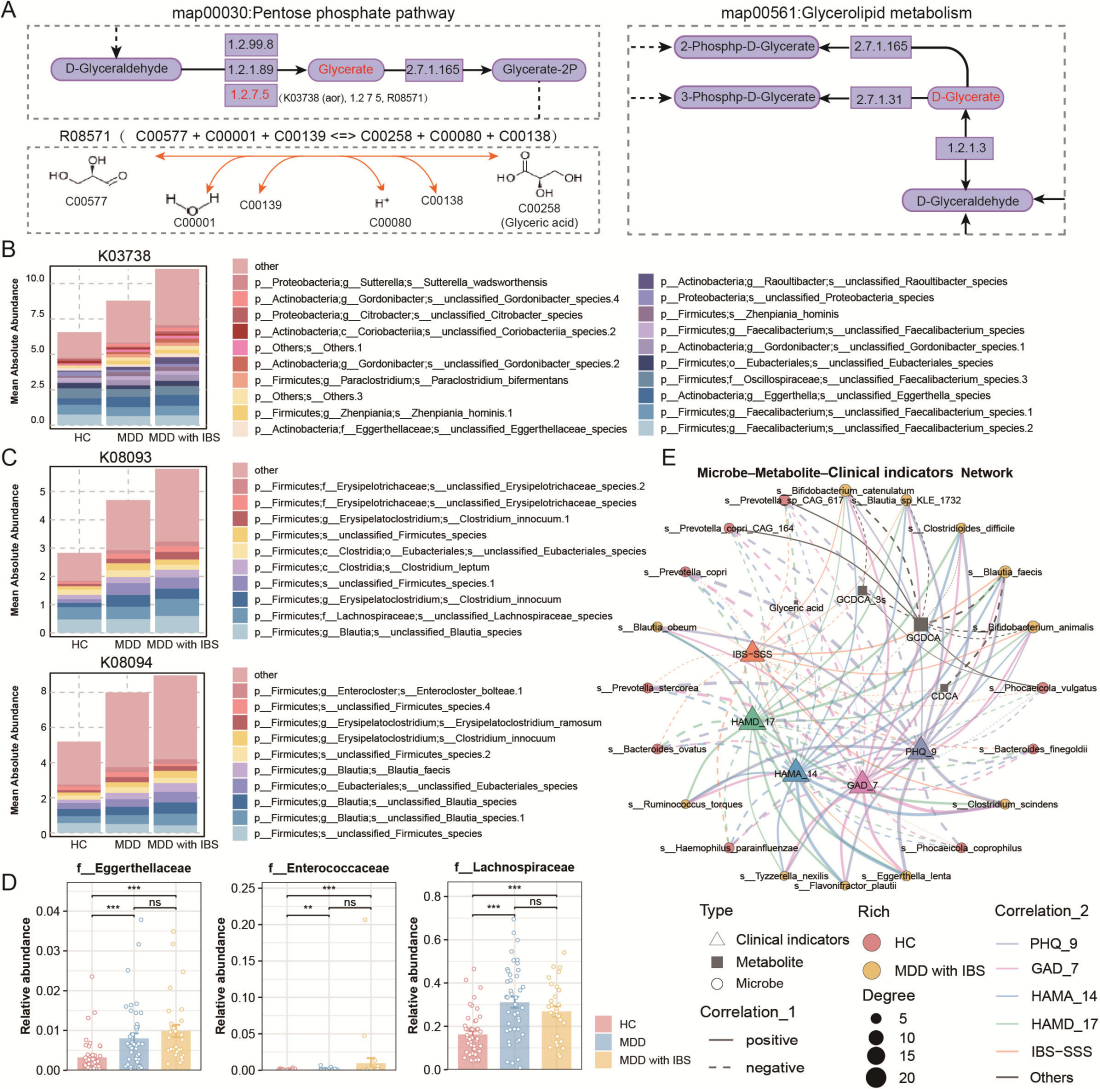
Functional annotation and differential metabolite analysis in the MDD with IBS group. (**A**) Schematic representation of glyceric acid metabolism in the KEGG pentose phosphate pathway (map00030) and glycerolipid metabolism pathway (map00561). In the pentose phosphate pathway, reaction EC1.2.7.5 is catalyzed by aldehyde ferredoxin oxidoreductase (AOR), encoded by KO gene K03738, with the reaction number R08571 and the reaction equation shown at the bottom left. (**B**) Bar plot showing the top 20 species contributing to KO gene K03738 across groups by mean absolute contribution. (**C**) Bar plot showing the top 10 species contributing to KO genes K08093 and K08094 across groups by mean absolute contribution. (**D**) Differential KO genes K08093, K08094, and others specific to the MDD with IBS group in the pentose phosphate pathway (map00030) are primarily derived from the *Firmicutes* families *Eggerthellaceae*, *Enterococcaceae*, and *Lachnospiraceae*. Their relative abundances are displayed as bar plots with overlaid dot plots. (**E**) Integrated correlation network among the top 20 significantly altered microbes, clinical indicators (emotional state and IBS-SSS scores), and key differential metabolites (including four bile acids and glyceric acid) in the MDD with IBS group. Statistical significance: ns, *P* ＞ 0.05; *, *P* < 0.05; **, *P* < 0.01; ***, *P* < 0.001.

To further investigate the potential impact of microbial functional alterations on host metabolism, we performed integrative functional annotation and metabolite analysis in the MDD with IBS group. The differential metabolite glyceric acid was found to be involved in several key KO-enriched pathways, including the pentose phosphate pathway (map00030), glycerolipid metabolism (map00561), and carbon metabolism (map01200). The pentose phosphate pathway is a part of carbon metabolism, and glyceric acid is regulated by several enzymes, such as EC1.2.1.98, EC1.2.99.8, and EC1.2.7.5. The KO gene K03738, which encodes aldehyde ferredoxin oxidoreductase that catalyzes the EC1.2.7.5 reaction, was significantly upregulated in the MDD with IBS group (Fig. 6A). Taxonomic AOR (K03738) revealed that its primary microbial sources were the phyla *Firmicutes* and *Actinobacteria*, with some sequences only annotated at the other level, including *Eggerthella* and *Oscillospiraceae*. These taxa exhibited significant differential abundance in the MDD with IBS group (Fig. 6B). Further analysis of other key differentially expressed KO genes within this pathway (K08093 and K08094) indicated that they were predominantly derived from *Firmicutes* (Fig. 6C), notably from the families *Eggerthellaceae*, *Enterococcaceae*, and *Lachnospiraceae*, which also showed significant shifts in abundance (Fig. 6D). Collectively, these findings suggest that multiple functionally relevant genes implicated in glyceric acid-associated metabolic pathways originate from gut microbial taxa that differ significantly in abundance in MDD with IBS.

Lastly, an integrated network was constructed to explore the correlations among the top 20 significantly altered microbes, clinical indicators (emotional state and IBS-SSS scores), and key metabolites (including four bile acids and glyceric acid) in the MDD with IBS group. Several differential microbes showed significant correlations with bile acid levels and clinical scores, whereas glyceric acid exhibited generally weak correlations with these variables (Fig. 6E).

## DISCUSSION

In this study, multi-omics analyses revealed that, compared to patients with MDD alone, those with MDD with IBS exhibited more severe anxiety and depressive symptoms, accompanied by alterations in gut microbiota composition, activation of functional pathways, and disturbances in serum metabolites. These findings support the critical role of the “gut-brain axis” in the comorbidity of MDD and IBS and suggest that IBS may exacerbate the progression of MDD via microbiota-metabolite mediated pathways.

Our research findings show that patients with MDD with IBS are more anxious and depressed than those with MDD alone. The HAMD-17 and HAMA-14 scores of patients with MDD with IBS are higher, but there are no significant differences in PHQ-9 and GAD-7 between the two groups. This might be due to the fact that the former is a clinical rating scale, while the latter is a concise self-assessment scale. It is common for the severity of depression, as assessed by physician rating and self-rating, to be inconsistent (39), and self-assessment scales rely on the patient’s self-awareness and honesty. Additionally, PHQ-9 is more comprehensive in assessing cognitive symptoms, such as a sense of worthlessness, while HAMD-17 is more detailed in evaluating physical symptoms, such as sleep and behavioral manifestations.

Our results showed that, relative to HC, both MDD and MDD with IBS groups demonstrated significantly increased Shannon and Chao1 indices, indicating enhanced species richness and diversity. However, the elevated Simpson index suggested a reduction in community evenness with a dominance of certain taxa. Beta diversity analysis revealed no significant difference in overall gut microbial structure between the two disease groups, indicating a broadly similar microbial community composition in MDD and MDD with IBS patients. This phenomenon implies that disease states may induce abnormal increases in microbial richness alongside decreased evenness, reflecting a dysbiotic rather than a healthy diversity state.

Further analysis demonstrated that both MDD and MDD with IBS groups had a significantly higher abundance of the phylum *Firmicutes* compared to HC, in which *Bacteroidota* predominated. As two key phyla maintaining intestinal homeostasis and metabolic balance, an altered *Firmicutes*-to-*Bacteroidota* ratio is often regarded as a hallmark of gut ecosystem disruption (40–43). Previous investigations have established associations between these two bacterial phyla and various disorders, including autoimmune diseases (17), metabolic diseases (2), as well as psychiatric diseases (44). Some studies have reported upregulation of these phyla in patients with MDD and functional gastrointestinal disorders (45, 46). Notably, the phylum *Actinobacteria* was significantly enriched in the MDD with IBS group. This phylum is closely associated with immune regulation and bile acid metabolism. For instance, *Eggerthella* was found to be increased in depression and anxiety cases across multiple studies, consistent with our findings (15, 47). *Eggerthella* may contribute to mood regulation by influencing host tryptophan metabolism or the synthesis of neuroactive compounds such as γ-aminobutyric acid (GABA) (48, 49). Additionally, earlier research identified gut bacterial taxa, including *Subdoligranulum*, *Coprococcus*, and *Ruminococcaceae*, as linked to major depressive disorder (47).

Although the gut microbial structures of the MDD and MDD with IBS groups were similar, functional gene and KEGG pathway analyses revealed more pronounced metabolic abnormalities under the comorbid condition, particularly exhibiting stronger functional activity in key pathways such as carbon, lipid, and amino acid metabolism. These alterations were significantly associated with patients’ emotional function and gastrointestinal symptom scores, suggesting that gut microbial metabolic function may mediate the interaction between emotional and somatic symptoms through the gut-brain axis, highlighting its importance in MDD and its comorbid states.

Through targeted metabolomics analysis, we identified several metabolites that exhibited significant differences in the MDD with IBS group. Firstly, we observed a marked decrease in branched-chain amino acids (BCAAs) in both the MDD and MDD with IBS groups. As precursors of key neurotransmitters, BCAAs compete with aromatic amino acids for blood-brain barrier transport, potentially limiting neurotransmitter synthesis (50, 51). While lower BCAA levels have been linked to MDD and proposed as biomarkers (52), a bidirectional Mendelian randomization study suggests that elevated BCAAs may actually increase MDD risk, indicating a complex relationship (53). Secondly, among the metabolites that showed significant differences only in the comparison between the MDD with IBS and HC groups, glutaconic acid and glyceric acid were significantly upregulated in the MDD with IBS group, while the three bile acids were significantly downregulated. At sufficiently high concentrations, both glutaconic acid and glyceric acid can act as metabolic toxins. Additionally, glutaconic acid exhibits excitotoxicity, which can damage neurons and induce apoptosis in immature oligodendrocytes (54). Several studies have demonstrated that a reciprocal regulation exists between microorganisms and BAs, with the bile acid-gut microbiota axis playing a crucial role in the pathogenesis and progression of IBS (16, 55–57). Among the serum metabolites identified, significant changes in BA levels were observed exclusively in the MDD with IBS group, suggesting that alterations in serum BA levels in MDD with IBS patients may result from interactions with the gut microbiota. Recent studies have also discovered neurotransmitters conjugated to BAs, like GABA and tyrosine conjugated BAs. In patients with ulcerative colitis pouchitis, their levels change, as seen in metabolomic analysis of J-pouch contents during pouchitis (58, 59). And changes in GABA-conjugated BAs were also detected in the brain. Given GABA and tyrosine’s neuroactive nature, their conjugated BAs may impact mood regulation via the gut-brain axis, highlighting the complexity and importance of this axis in disease mechanisms. Finally, we also observed a significant downregulation of N-acetylglutamine, imidazolepropionic acid, acetic acid, and N-acetylaspartic acid in the MDD with IBS group compared to the MDD group. NAG is an acetylated derivative of glutamine (60). Previous studies have demonstrated that glutamine possesses clinical significance in immune regulation and gastrointestinal disorder treatment (61). Early studies reported that patients with intestinal disorders excrete imidazolepropionic acid (62); however, in our study, its serum levels were decreased, suggesting that the *in vivo* circulation mechanism of this metabolite requires further investigation. In line with this, Wu et al. reported decreased fecal acetate levels in mice exhibiting depressive symptoms (63). Furthermore, numerous clinical investigations have consistently reported decreased NAA content in the brains of depressed patients (64, 65), suggesting potential neuronal damage. Collectively, our research results indicate that the differential metabolites identified in patients with MDD with IBS are closely related to the gut-microbiome-brain axis. These metabolites act as key “messengers” connecting the intestinal microbial activity with the central nervous system function. This emphasizes that targeting this axis and its associated metabolic pathways for intervention may be crucial in unraveling the pathogenesis of the comorbidity of MDD and IBS and in developing novel therapeutic strategies.

Emotional state may influence both the blood metabolome and gut microbiota. Previous studies have reported associations between emotional function and gut microbial composition as well as metabolites (66, 67). In this study, no significant covariate effects were detected in separate analyses, possibly limited by the metabolite detection coverage, which encompassed only approximately 200 metabolites and thus could not comprehensively reflect the complexity of the blood metabolome (68). We further conducted correlation analyses between significantly altered metabolites and microbiota in the MDD with IBS group, identifying significant associations between primary bile acids such as CDCA and GCDCA and multiple differential taxa. Among negatively correlated taxa, *Gordonibacter pamelaeae* can produce specific bile acid derivatives that regulate TH17 cells (69). Three positively correlated taxa belonged to unnamed Bacteroides, many members of which, such as *Bacteroides fragilis* and *Bacteroides thetaiotaomicron*, possess bile salt hydrolase activity and participate in bile acid metabolism (70–72). These findings suggest that the comorbid state may trigger specific metabolite–microbiota interaction patterns. Moreover, among differential taxa shared by both disease groups, several strains previously reported to be directly involved in bile acid metabolism were identified. For example, *Eggerthella lenta* modifies bile acid structures via oxidoreductases and decarboxylases, while *Clostridium scindens* harbors the 7α-dehydroxylase-encoding gene cluster, enabling conversion of primary to secondary bile acids (70). These results further support the hypothesis that bile acids act as potential key mediators in the gut-brain axis. Recent studies have further revealed that the BAs driven by the gut microbiome exhibit diversity. For instance, Nie et al. identified several bile acids produced by microorganisms (73). This study has certain limitations in covering the entire panorama of the interaction between BAs and the microbiome, and it is also suggested that in-depth exploration of the mechanism by which the microbiome regulates the diversity of BAs will be the core direction for future research on the role of the intestinal-microbiome-BAs axis in MDD with IBS. Additionally, glyceric acid was significantly elevated in the MDD with IBS group and was involved in multiple significantly enriched metabolic pathways. Source-tracking analysis revealed that key KO genes related to glyceric acid synthesis were significantly upregulated in this comorbid group, primarily derived from specific taxa within the phyla *Actinobacteria* and *Firmicutes*, such as *Eggerthella* and *Oscillospiraceae*. These bacterial communities mainly participate in the glycerol acid production process through their own metabolic activities, jointly providing microbial-level support for the elevated glycerol acid levels in the MDD with IBS group. Network analysis revealed generally weak associations between major differential taxa and both clinical indicators and glyceric acid levels, suggesting that the alteration in glyceric acid may not be directly driven by a single microbial species but rather by functional changes resulting from broader shifts in the gut microbial community structure.

This study identifies a concurrent dysregulation of bile acids and glyceric acid as a potential metabolic hallmark of MDD with IBS. On one hand, strains such as *Eggerthella lenta* and *Clostridium scindens* may promote the metabolism and depletion of primary bile acids. This may reduce the activation of TGR5/FXR, which may affect the integrity of the intestinal barrier, lead to the translocation of bacteria, and result in the occurrence of chronic inflammation (74). On the other hand, the accumulation of glyceric acid, potentially driven by gut microbial activity, may modulate the host oxidative stress response and GABAergic system indirectly by influencing NADPH production and competing for metabolic substrates. This is based on the proposed link between glyceric acid metabolism and NADPH homeostasis (75–78). Further experimental studies are required to validate these mechanisms.

It is worth noting that although we observed distinct differences between the MDD and MDD with IBS groups at both metagenomic and metabolomic levels, the differences in beta diversity and differential microbial taxa were relatively modest. This may suggest that IBS exerts its impact on MDD primarily through functional disruptions of the gut microbiota, rather than through large-scale changes in microbial composition. Alternatively, the weak group-level differences may be attributable to limited sample size or heterogeneity among IBS subtypes.

In conclusion, our findings highlight the potential role of gut microbiota and their metabolites in the pathophysiology of MDD with IBS and suggest that gut-targeted therapies—such as probiotics, prebiotics, or fecal microbiota transplantation—may hold promise for this patient population. Moreover, the present study provides novel multi-omics evidence that may contribute to the future classification of MDD subtypes and development of individualized therapeutic strategies.

## Data Availability

The raw metagenomic sequencing data have been deposited in the National Genomics Data Center (NGDC) (https://ngdc.cncb.ac.cn/) under accession number PRJCA043176.

## SUPPLEMENTAL MATERIAL

Fig. S1 mbio.02447-25-s0001.tif KEGG pathway enrichment analysis in HC vs. MDD and HC vs. MDD with IBS groups.

Fig. S2 mbio.02447-25-s0002.tif Correlation between enriched pathways and clinical indices.

Legends mbio.02447-25-s0003.docx Supplemental figure legends.

Table S1 mbio.02447-25-s0004.xls Summary of sequencing data and microbial read proportions for each sample.

Table S2 mbio.02447-25-s0005.xlsx Significantly different microbial taxa between groups based on metagenomic LEfSe analysis.

Table S3 mbio.02447-25-s0006.xlsx Normalized serum metabolite data.

Table S4 mbio.02447-25-s0007.xlsx Significantly different metabolites between groups.

ASM does not own the copyrights to Supplemental Material that may be linked to, or accessed through, an article. The authors have granted ASM a non-exclusive, world-wide license to publish the Supplemental Material files. Please contact the corresponding author directly for reuse.

## References

[1] Herrman H, Kieling C, McGorry P, Horton R, Sargent J, Patel V. 2019. Reducing the global burden of depression: a Lancet-World Psychiatric Association Commission. Lancet 393:e42–e43. doi:10.1016/S0140-6736(18)32408-5 30482607

[2] Longstreth GF, Thompson WG, Chey WD, Houghton LA, Mearin F, Spiller RC. 2006. Functional bowel disorders. Gastroenterology 130:1480–1491. doi:10.1053/j.gastro.2005.11.061 16678561

[3] Simpson CA, Mu A, Haslam N, Schwartz OS, Simmons JG. 2020. Feeling down? A systematic review of the gut microbiota in anxiety/depression and irritable bowel syndrome. J Affect Disord 266:429–446. doi:10.1016/j.jad.2020.01.124 32056910

[4] Staudacher HM, Black CJ, Teasdale SB, Mikocka-Walus A, Keefer L. 2023. Irritable bowel syndrome and mental health comorbidity — approach to multidisciplinary management. Nat Rev Gastroenterol Hepatol 20:582–596. doi:10.1038/s41575-023-00794-z 37268741 PMC10237074

[5] Lee Y-T, Hu L-Y, Shen C-C, Huang M-W, Tsai S-J, Yang AC, Hu C-K, Perng C-L, Huang Y-S, Hung J-H. 2015. Risk of psychiatric disorders following irritable bowel syndrome: a nationwide population-based cohort study. PLoS One 10:e0133283. doi:10.1371/journal.pone.0133283 26222511 PMC4519183

[6] Wohleb ES, Terwilliger R, Duman CH, Duman RS. 2018. Stress-induced neuronal colony stimulating factor 1 provokes microglia-mediated neuronal remodeling and depressive-like behavior. Biol Psychiatry 83:38–49. doi:10.1016/j.biopsych.2017.05.026 28697890 PMC6506225

[7] Goodoory VC, Mikocka-Walus A, Yiannakou Y, Houghton LA, Black CJ, Ford AC. 2021. Impact of psychological comorbidity on the prognosis of irritable bowel syndrome. Am J Gastroenterol 116:1485–1494. doi:10.14309/ajg.0000000000001247 33840729

[8] Farzaneh N, Ghobakhlou M, Moghimi-Dehkordi B, Naderi N, Fadai F. 2012. Evaluation of psychological aspects among subtypes of irritable bowel syndrome. Indian J Psychol Med 34:144–148. doi:10.4103/0253-7176.101780 23162190 PMC3498777

[9] Mohammed AA, Moustafa HA, Nour-Eldein H, Saudi RA. 2021. Association of anxiety-depressive disorders with irritable bowel syndrome among patients attending a rural family practice center: a comparative cross-sectional study. Gen Psychiatr 34:e100553. doi:10.1136/gpsych-2021-100553 34970639 PMC8671974

[10] Han L, Zhao L, Zhou Y, Yang C, Xiong T, Lu L, Deng Y, Luo W, Chen Y, Qiu Q, et al.. 2022. Altered metabolome and microbiome features provide clues in understanding irritable bowel syndrome and depression comorbidity. ISME J 16:983–996. doi:10.1038/s41396-021-01123-5 34750528 PMC8940891

[11] Liu Y, Zhang L, Wang X, Wang Z, Zhang J, Jiang R, Wang X, Wang K, Liu Z, Xia Z, Xu Z, Nie Y, Lv X, Wu X, Zhu H, Duan L. 2016. Similar fecal microbiota signatures in patients with diarrhea-predominant irritable bowel syndrome and patients with depression. Clin Gastroenterol Hepatol 14:1602–1611. doi:10.1016/j.cgh.2016.05.033 27266978

[12] Zhao J, Li X, Wang X, Wang X, Hao X, Li Z, Zhu L. 2024. The value of PHQ-9 and GAD-7 for screening emotional disorders in IBS-D and the specificity of the gut flora associated with emotional comorbidity: preliminary findings. Neuropsychiatr Dis Treat 20:2145–2158. doi:10.2147/NDT.S486784 39564595 PMC11573876

[13] Liu Q, Fang W, Zheng P, Xie S, Jiang X, Luo W, Han L, Zhao L, Lu L, Zhai L, Yu DJ, Yang W, Lin C, Fang X, Bian Z. 2025. Multi-kingdom microbiota analysis reveals bacteria-viral interplay in IBS with depression and anxiety. NPJ Biofilms Microbiomes 11:129. doi:10.1038/s41522-025-00760-4 40617850 PMC12228763

[14] Jiang H, Ling Z, Zhang Y, Mao H, Ma Z, Yin Y, Wang W, Tang W, Tan Z, Shi J, Li L, Ruan B. 2015. Altered fecal microbiota composition in patients with major depressive disorder. Brain Behav Immun 48:186–194. doi:10.1016/j.bbi.2015.03.016 25882912

[15] Simpson CA, Diaz-Arteche C, Eliby D, Schwartz OS, Simmons JG, Cowan CSM. 2021. The gut microbiota in anxiety and depression - A systematic review. Clin Psychol Rev 83:101943. doi:10.1016/j.cpr.2020.101943 33271426

[16] Raskov H, Burcharth J, Pommergaard H-C, Rosenberg J. 2016. Irritable bowel syndrome, the microbiota and the gut-brain axis. Gut Microbes 7:365–383. doi:10.1080/19490976.2016.1218585 27472486 PMC5046167

[17] Yang L, Xiang Z, Zou J, Zhang Y, Ni Y, Yang J. 2022. Comprehensive analysis of the relationships between the gut microbiota and fecal metabolome in individuals with primary Sjogren's syndrome by 16S rRNA sequencing and LC-MS-based metabolomics. Front Immunol 13. doi:10.3389/fimmu.2022.874021 PMC9130595 35634334

[18] Zheng H, Zhao Q, Chen J, Lu J, Li Y, Gao H. 2023. Gastrointestinal microbiome of ARDS patients induces neuroinflammation and cognitive impairment in mice. J Neuroinflammation 20:166. doi:10.1186/s12974-023-02825-7 37454113 PMC10349492

[19] Mokkala K, Paulin N, Houttu N, Koivuniemi E, Pellonperä O, Khan S, Pietilä S, Tertti K, Elo LL, Laitinen K. 2021. Metagenomics analysis of gut microbiota in response to diet intervention and gestational diabetes in overweight and obese women: a randomised, double-blind, placebo-controlled clinical trial. Gut 70:309–318. doi:10.1136/gutjnl-2020-321643 32839200

[20] Liu L, Wang H, Chen X, Zhang Y, Zhang H, Xie P. 2023. Gut microbiota and its metabolites in depression: from pathogenesis to treatment. EBioMedicine 90:104527. doi:10.1016/j.ebiom.2023.104527 36963238 PMC10051028

[21] Yang J, Palmiotti A, Kuipers F. 2021. Emerging roles of bile acids in control of intestinal functions. Curr Opin Clin Nutr Metab Care 24:127–133. doi:10.1097/MCO.0000000000000709 33075001

[22] Fu L, Wang M, Li D, Ma S, Zhang F, Zheng L. 2025. Microbial metabolites short chain fatty acids, tight junction, gap junction, and reproduction: a review. Front Cell Dev Biol 13:1624415. doi:10.3389/fcell.2025.1624415 40917749 PMC12411448

[23] Babenkova P, Gureev A, Sadovnikova I, Burakova I, Smirnova Y, Pogorelova S, Morozova P, Gribovskaya V, Adzhemian D, Syromyatnikov M. 2025. Changes in L-carnitine metabolism affect the gut microbiome and influence sexual behavior through the gut-testis axis. Microorganisms 13:1751. doi:10.3390/microorganisms13081751 40871254 PMC12388078

[24] Zhou M, Fan Y, Xu L, Yu Z, Wang S, Xu H, Zhang J, Zhang L, Liu W, Wu L, Yu J, Yao H, Wang J, Gao R. 2023. Microbiome and tryptophan metabolomics analysis in adolescent depression: roles of the gut microbiota in the regulation of tryptophan-derived neurotransmitters and behaviors in human and mice. Microbiome 11:145. doi:10.1186/s40168-023-01589-9 37386523 PMC10311725

[25] Zu X, Xin J, Xie H, Xu X, Shen Y, Wang J, Tian S, Wen Y, Li H, Yang J, Fang Y. 2024. Characteristics of gut microbiota and metabolic phenotype in patients with major depressive disorder based on multi-omics analysis. J Affect Disord 344:563–576. doi:10.1016/j.jad.2023.10.104 37863362

[26] Mayneris-Perxachs J, Castells-Nobau A, Arnoriaga-Rodríguez M, Martin M, de la Vega-Correa L, Zapata C, Burokas A, Blasco G, Coll C, Escrichs A, et al.. 2022. Microbiota alterations in proline metabolism impact depression. Cell Metab 34:681–701. doi:10.1016/j.cmet.2022.04.001 35508109

[27] Lecrubier Y, Sheehan DV, Weiller E, Amorim P, Bonora I, Sheehan KH, Janavs J, Dunbar GC. 1997. The MINI International Neuropsychiatric Interview (MINI). A short diagnostic structured interview: reliability and validity according to the CIDI. Eur Psychiatr 12:224–231. doi:10.1016/S0924-9338(97)83296-8

[28] Francis CY, Morris J, Whorwell PJ. 1997. The irritable bowel severity scoring system: a simple method of monitoring irritable bowel syndrome and its progress. Aliment Pharmacol Ther 11:395–402. doi:10.1046/j.1365-2036.1997.142318000.x 9146781

[29] Hamilton M. 1960. A rating scale for depression. J Neurol Neurosurg Psychiatry 23:56–62. doi:10.1136/jnnp.23.1.56 14399272 PMC495331

[30] Hamilton M. 1959. The assessment of anxiety-states by rating. Br J Med Psychol 32:50–55. doi:10.1111/j.2044-8341.1959.tb00467.x 13638508

[31] Kroenke K, Spitzer RL, Williams JBW. 2001. The PHQ-9: validity of a brief depression severity measure. J Gen Intern Med 16:606–613. doi:10.1046/j.1525-1497.2001.016009606.x 11556941 PMC1495268

[32] Spitzer RL, Kroenke K, Williams JBW, Löwe B. 2006. A brief measure for assessing generalized anxiety disorder: the GAD-7. Arch Intern Med 166:1092–1097. doi:10.1001/archinte.166.10.1092 16717171

[33] Xie G, Wang L, Chen T, Zhou K, Zhang Z, Li J, Sun B, Guo Y, Wang X, Wang Y, Zhang H, Liu P, Nicholson JK, Ge W, Jia W. 2021. A metabolite array technology for precision medicine. Anal Chem 93:5709–5717. doi:10.1021/acs.analchem.0c04686 33797874

[34] Panos A, Mavridis D. 2020. TableOne: an online web application and R package for summarising and visualising data. Evid Based Ment Health 23:127–130. doi:10.1136/ebmental-2020-300162 32665250 PMC10231609

[35] Dixon P. 2003. VEGAN, a package of R functions for community ecology. J Vegetation Sci 14:927–930. doi:10.1111/j.1654-1103.2003.tb02228.x

[36] Liu C, Cui YM, Li XZ, Yao MJ. 2021. microeco: an R package for data mining in microbial community ecology. FEMS Microbiol Ecol 97. doi:10.1093/femsec/fiaa255 33332530

[37] Peng C, Chen Q, Tan S, Shen X, Jiang C. 2024. Generalized reporter score-based enrichment analysis for omics data. Brief Bioinform 25:bbae116. doi:10.1093/bib/bbae116 38546324 PMC10976918

[38] Culhane AC, Thioulouse J, Perrière G, Higgins DG. 2005. MADE4: an R package for multivariate analysis of gene expression data. Bioinformatics 21:2789–2790. doi:10.1093/bioinformatics/bti394 15797915

[39] Cuijpers P, Li J, Hofmann SG, Andersson G. 2010. Self-reported versus clinician-rated symptoms of depression as outcome measures in psychotherapy research on depression: a meta-analysis. Clin Psychol Rev 30:768–778. doi:10.1016/j.cpr.2010.06.001 20619943

[40] Magne F, Gotteland M, Gauthier L, Zazueta A, Pesoa S, Navarrete P, Balamurugan R. 2020. The Firmicutes/Bacteroidetes ratio: a relevant marker of gut dysbiosis in obese patients? Nutrients 12:1474. doi:10.3390/nu12051474 32438689 PMC7285218

[41] Houtman TA, Eckermann HA, Smidt H, de Weerth C. 2022. Gut microbiota and BMI throughout childhood: the role of Firmicutes, bacteroidetes, and short-chain fatty acid producers. Sci Rep 12:3140. doi:10.1038/s41598-022-07176-6 35210542 PMC8873392

[42] Nkosi BVZ, Padayachee T, Gront D, Nelson DR, Syed K. 2022. Contrasting health effects of Bacteroidetes and Firmicutes lies in their genomes: analysis of P450s, ferredoxins, and secondary metabolite clusters. Int J Mol Sci 23:5057. doi:10.3390/ijms23095057 35563448 PMC9100364

[43] Mantovani A, Longo L, Thoen RU, Rampelotto PH, Salinas R, Guerreiro GTS, Álvares-da-Silva MR. 2024. Firmicutes/Bacteroidetes and Firmicutes/Proteobacteria ratios are associated with worse prognosis in a cohort of Latin American patients with cirrhosis. Clinics (Sao Paulo) 79:100471. doi:10.1016/j.clinsp.2024.100471 39098143 PMC11345307

[44] Chung Y-CE, Chen H-C, Chou H-CL, Chen I-M, Lee M-S, Chuang L-C, Liu Y-W, Lu M-L, Chen C-H, Wu C-S, Huang M-C, Liao S-C, Ni Y-H, Lai M-S, Shih W-L, Kuo P-H. 2019. Exploration of microbiota targets for major depressive disorder and mood related traits. J Psychiatr Res 111:74–82. doi:10.1016/j.jpsychires.2019.01.016 30685565

[45] Labus JS, Hollister EB, Jacobs J, Kirbach K, Oezguen N, Gupta A, Acosta J, Luna RA, Aagaard K, Versalovic J, Savidge T, Hsiao E, Tillisch K, Mayer EA. 2017. Differences in gut microbial composition correlate with regional brain volumes in irritable bowel syndrome. Microbiome 5:49. doi:10.1186/s40168-017-0260-z 28457228 PMC5410709

[46] Zheng P, Zeng B, Zhou C, Liu M, Fang Z, Xu X, Zeng L, Chen J, Fan S, Du X, Zhang X, Yang D, Yang Y, Meng H, Li W, Melgiri ND, Licinio J, Wei H, Xie P. 2016. Gut microbiome remodeling induces depressive-like behaviors through a pathway mediated by the host’s metabolism. Mol Psychiatry 21:786–796. doi:10.1038/mp.2016.44 27067014

[47] Radjabzadeh D, Bosch JA, Uitterlinden AG, Zwinderman AH, Ikram MA, van Meurs JBJ, Luik AI, Nieuwdorp M, Lok A, van Duijn CM, Kraaij R, Amin N. 2022. Gut microbiome-wide association study of depressive symptoms. Nat Commun 13:7128. doi:10.1038/s41467-022-34502-3 36473852 PMC9726982

[48] Francavilla M, Facchetti S, Demartini C, Zanaboni AM, Amoroso C, Bottiroli S, Tassorelli C, Greco R. 2024. A narrative review of intestinal microbiota's impact on migraine with psychopathologies. Int J Mol Sci 25:6655. doi:10.3390/ijms25126655 38928361 PMC11203823

[49] McGuinness AJ, Davis JA, Dawson SL, Loughman A, Collier F, O’Hely M, Simpson CA, Green J, Marx W, Hair C, Guest G, Mohebbi M, Berk M, Stupart D, Watters D, Jacka FN. 2022. A systematic review of gut microbiota composition in observational studies of major depressive disorder, bipolar disorder and schizophrenia. Mol Psychiatry 27:1920–1935. doi:10.1038/s41380-022-01456-3 35194166 PMC9126816

[50] Neinast M, Murashige D, Arany Z. 2019. Branched chain amino acids. Annu Rev Physiol 81:139–164. doi:10.1146/annurev-physiol-020518-114455 30485760 PMC6536377

[51] Zakaria F, Akhtar MT, Wan Norhamidah WI, Noraini AB, Muhamad A, Shohaimi S, Maulidiani, Ahmad H, Ismail IS, Ismail NH, Shaari K. 2023. Centella asiatica (L.) Urb. extract ameliorates branched-chain amino acid (BCAA) metabolism in acute reserpine-induced stress zebrafish model via ^1^H Nuclear Magnetic Resonance (NMR)-based metabolomics approach. Comp Biochem Physiol C Toxicol Pharmacol 264:109501. doi:10.1016/j.cbpc.2022.109501 36336330

[52] Baranyi A, Amouzadeh-Ghadikolai O, von Lewinski D, Rothenhäusler H-B, Theokas S, Robier C, Mangge H, Reicht G, Hlade P, Meinitzer A. 2016. Branched-chain amino acids as new biomarkers of major depression - a novel neurobiology of mood disorder. PLoS One 11:e0160542. doi:10.1371/journal.pone.0160542 27490818 PMC4973973

[53] Ma Z, Zhang R, Yuan D, Yu C, Baranova A, Cao H, Zhang F. 2025. Association of branched-chain amino acids with major depressive disorder: a bidirectional Mendelian randomization study. J Affect Disord 379:467–472. doi:10.1016/j.jad.2025.03.032 40081595

[54] Gerstner B, Gratopp A, Marcinkowski M, Sifringer M, Obladen M, Bührer C. 2005. Glutaric acid and its metabolites cause apoptosis in immature oligodendrocytes: a novel mechanism of white matter degeneration in glutaryl-CoA dehydrogenase deficiency. Pediatr Res 57:771–776. doi:10.1203/01.PDR.0000157727.21503.8D 15774829

[55] Wong BS, Camilleri M, Carlson P, McKinzie S, Busciglio I, Bondar O, Dyer RB, Lamsam J, Zinsmeister AR. 2012. Increased bile acid biosynthesis is associated with irritable bowel syndrome with diarrhea. Clin Gastroenterol Hepatol 10:1009–15. doi:10.1016/j.cgh.2012.05.006 22610000 PMC3565429

[56] Wei W, Wang HF, Zhang Y, Zhang YL, Niu BY, Yao SK. 2020. Altered metabolism of bile acids correlates with clinical parameters and the gut microbiota in patients with diarrhea-predominant irritable bowel syndrome. World J Gastroenterol 26:7153–7172. doi:10.3748/wjg.v26.i45.7153 33362374 PMC7723672

[57] Deng X, Xiao L, Luo M, Xie P, Xiong L. 2023. Intestinal crosstalk between bile acids and microbiota in irritable bowel syndrome. J Gastroenterol Hepatol 38:1072–1082. doi:10.1111/jgh.16159 36869260

[58] Mullowney MW, Fiebig A, Schnizlein MK, McMillin M, Rose AR, Koval J, Rubin D, Dalal S, Sogin ML, Chang EB, Sidebottom AM, Crosson S. 2024. Microbially catalyzed conjugation of GABA and tyramine to bile acids. J Bacteriol 206:e0042623. doi:10.1128/jb.00426-23 38174933 PMC10810215

[59] Romero-Ramírez L, Mey J. 2024. Emerging roles of bile acids and TGR5 in the central nervous system: molecular functions and therapeutic implications. Int J Mol Sci 25:9279. doi:10.3390/ijms25179279 39273226 PMC11395147

[60] Li X, Wang T, Zhang D, Li H, Shen H, Ding X, Chen G. 2018. Andrographolide ameliorates intracerebral hemorrhage induced secondary brain injury by inhibiting neuroinflammation induction. Neuropharmacology 141:305–315. doi:10.1016/j.neuropharm.2018.09.015 30218674

[61] López-Pedrosa JM, Manzano M, Baxter JH, Rueda R. 2007. N-acetyl-L-glutamine, a liquid-stable source of glutamine, partially prevents changes in body weight and on intestinal immunity induced by protein energy malnutrition in pigs. Dig Dis Sci 52:650–658. doi:10.1007/s10620-006-9500-y 17253138

[62] van der Heiden C, Wadman SK, de Bree PK, Wauters EA. 1972. Increased urinary imidazolepropionic acid, N-acetylhistamine and other imidazole compounds in patients with intestinal disorders. Clin Chim Acta 39:201–214. doi:10.1016/0009-8981(72)90317-8 5038749

[63] Wu M, Tian T, Mao Q, Zou T, Zhou CJ, Xie J, Chen JJ. 2020. Associations between disordered gut microbiota and changes of neurotransmitters and short-chain fatty acids in depressed mice. Transl Psychiatry 10:350. doi:10.1038/s41398-020-01038-3 33067412 PMC7567879

[64] Jayaweera HK, Lagopoulos J, Duffy SL, Lewis SJG, Hermens DF, Norrie L, Hickie IB, Naismith SL. 2015. Spectroscopic markers of memory impairment, symptom severity and age of onset in older people with lifetime depression: discrete roles of N-acetyl aspartate and glutamate. J Affect Disord 183:31–38. doi:10.1016/j.jad.2015.04.023 26000754

[65] Tosun Ş, Tosun M, Akansel G, Gökbakan AM, Ünver H, Tural Ü. 2020. Proton magnetic resonance spectroscopic analysis of changes in brain metabolites following electroconvulsive therapy in patients with major depressive disorder. Int J Psychiatry Clin Pract 24:96–101. doi:10.1080/13651501.2019.1699118 31825726

[66] Jia M, Fan Y, Ma Q, Yang D, Wang Y, He X, Zhao B, Zhan X, Qi Z, Ren Y, Dong Z, Zhu F, Wang W, Gao Y, Ma X. 2024. Gut microbiota dysbiosis promotes cognitive impairment via bile acid metabolism in major depressive disorder. Transl Psychiatry 14:503. doi:10.1038/s41398-024-03211-4 39719433 PMC11668851

[67] Kim CS. 2024. Roles of diet-associated gut microbial metabolites on brain health: cell-to-cell interactions between gut bacteria and the central nervous system. Adv Nutr 15:100136. doi:10.1016/j.advnut.2023.10.008 38436218 PMC10694655

[68] Wishart DS. 2019. Metabolomics for investigating physiological and pathophysiological processes. Physiol Rev 99:1819–1875. doi:10.1152/physrev.00035.2018 31434538

[69] Paik D, Yao L, Zhang Y, Bae S, D’Agostino GD, Zhang M, Kim E, Franzosa EA, Avila-Pacheco J, Bisanz JE, Rakowski CK, Vlamakis H, Xavier RJ, Turnbaugh PJ, Longman RS, Krout MR, Clish CB, Rastinejad F, Huttenhower C, Huh JR, Devlin AS. 2022. Human gut bacteria produce ΤΗ17-modulating bile acid metabolites. Nature 603:907–912. doi:10.1038/s41586-022-04480-z 35296854 PMC9132548

[70] Cai J, Sun L, Gonzalez FJ. 2022. Gut microbiota-derived bile acids in intestinal immunity, inflammation, and tumorigenesis. Cell Host Microbe 30:289–300. doi:10.1016/j.chom.2022.02.004 35271802 PMC8923532

[71] McMillan AS, Foley MH, Perkins CE, Theriot CM. 2023. Loss of Bacteroides thetaiotaomicron bile acid altering enzymes impact bacterial fitness and the global metabolic transcriptome. bioRxiv:2023.06.27.546749. doi:10.1101/2023.06.27.546749 PMC10783122 38018975

[72] Sun L, Zhang Y, Cai J, Rimal B, Rocha ER, Coleman JP, Zhang C, Nichols RG, Luo Y, Kim B, Chen Y, Krausz KW, Harris CC, Patterson AD, Zhang Z, Takahashi S, Gonzalez FJ. 2023. Bile salt hydrolase in non-enterotoxigenic Bacteroides potentiates colorectal cancer. Nat Commun 14:755. doi:10.1038/s41467-023-36089-9 36765047 PMC9918522

[73] Nie Q, Luo X, Wang K, Ding Y, Jia S, Zhao Q, Li M, Zhang J, Zhuo Y, Lin J, Guo C, Zhang Z, Liu H, Zeng G, You J, Sun L, Lu H, Ma M, Jia Y, Zheng M-H, Pang Y, Qiao J, Jiang C. 2024. Gut symbionts alleviate MASH through a secondary bile acid biosynthetic pathway. Cell 187:2717–2734. doi:10.1016/j.cell.2024.03.034 38653239

[74] Fiorucci S, Mencarelli A, Palladino G, Cipriani S. 2009. Bile-acid-activated receptors: targeting TGR5 and farnesoid-X-receptor in lipid and glucose disorders. Trends Pharmacol Sci 30:570–580. doi:10.1016/j.tips.2009.08.001 19758712

[75] Stincone A, Prigione A, Cramer T, Wamelink MMC, Campbell K, Cheung E, Olin-Sandoval V, Grüning N-M, Krüger A, Tauqeer Alam M, Keller MA, Breitenbach M, Brindle KM, Rabinowitz JD, Ralser M. 2015. The return of metabolism: biochemistry and physiology of the pentose phosphate pathway. Biol Rev Camb Philos Soc 90:927–963. doi:10.1111/brv.12140 25243985 PMC4470864

[76] Sies H. 2015. Oxidative stress: a concept in redox biology and medicine. Redox Biol 4:180–183. doi:10.1016/j.redox.2015.01.002 25588755 PMC4309861

[77] Luscher B, Maguire JL, Rudolph U, Sibille E. 2023. GABA_A_ receptors as targets for treating affective and cognitive symptoms of depression. Trends Pharmacol Sci 44:586–600. doi:10.1016/j.tips.2023.06.009 37543478 PMC10511219

[78] Kim K, Yoon H. 2023. Gamma-aminobutyric acid signaling in damage response, metabolism, and disease. Int J Mol Sci 24:4584. doi:10.3390/ijms24054584 36902014 PMC10003236

