# Supplementary figures and images for: Gut microbiota dysbiosis and metabolic perturbations of bile/glyceric acids in major depressive disorder with IBS comorbidity
Source: mBio. 2025 Oct 7;16(11):e02447-25. doi: 10.1128/mbio.02447-25 (PMC12607870; doi:10.1128/mbio.02447-25)

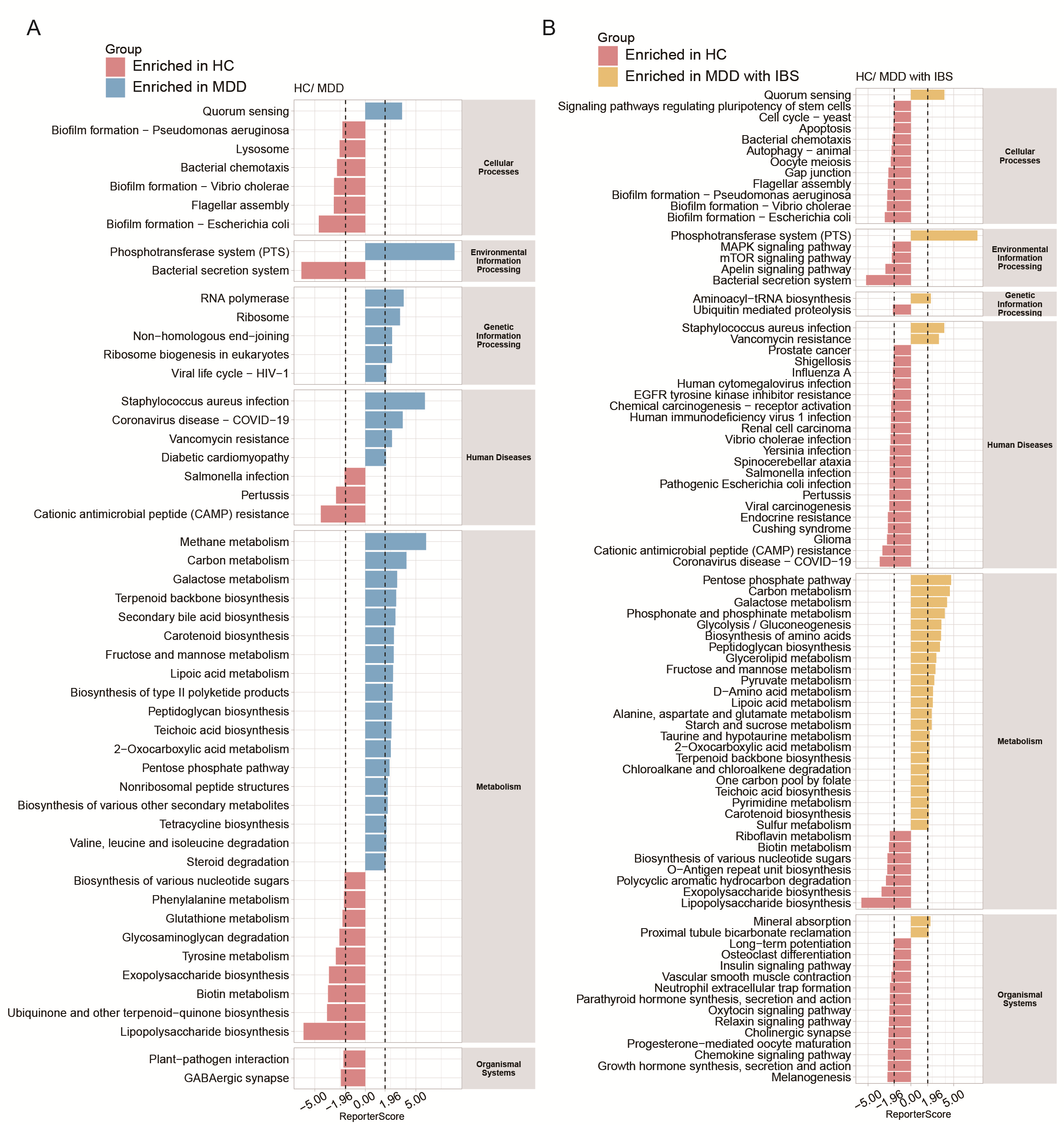

Supplement: Fig. S1 — KEGG pathway enrichment analysis in HC vs. MDD and HC vs. MDD with IBS groups. [file mbio.02447-25-s0001.tif]

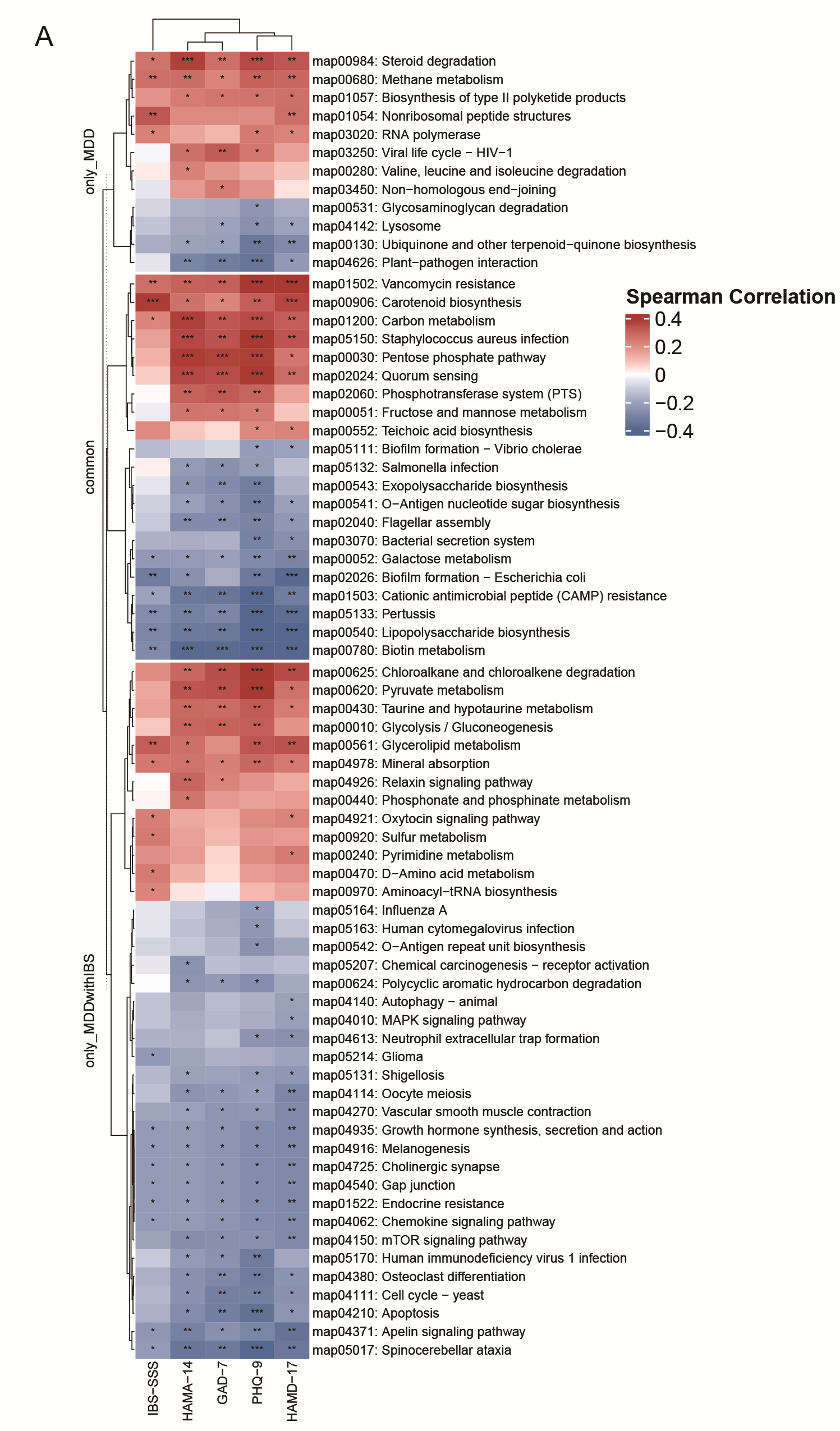

Supplement: Fig. S2 — Correlation between enriched pathways and clinical indices. [file mbio.02447-25-s0002.tif]
