# Supplementary material for: Gut microbiota dysbiosis and metabolic perturbations of bile/glyceric acids in major depressive disorder with IBS comorbidity
Source: mBio. 2025 Oct 7;16(11):e02447-25. doi: 10.1128/mbio.02447-25 (PMC12607870; doi:10.1128/mbio.02447-25)
Supplement: Legends — Supplemental figure legends. [file mbio.02447-25-s0003.docx]

**Supplementary figure 1. KEGG pathway enrichment analysis in HC vs. MDD and HC vs. MDD with IBS groups. A)** KEGG pathway enrichment analysis between HC and MDD groups based on reporter score (|ReporterScore | > 1.96). **B)** KEGG pathway enrichment analysis between HC and MDD with IBS groups based on reporter score (|ReporterScore | > 1.96). Pathway enrichment was conducted using the Generalized Reporter Score Analysis (GRSA) method

**Supplementary figure 2.** **Correlation between enriched pathways and clinical indices.** **A)** Spearman correlation heatmap between enriched pathways and emotional state, as well as IBS-SSS scores. Statistical significance: P < 0.05 (*), P < 0.01 (**), P < 0.001 (***).
